# Vertically Transmitted *Epichloë* Systemic Endophyte Enhances Drought Tolerance of *Achnatherum inebrians* Host Plants through Promoting Photosynthesis and Biomass Accumulation

**DOI:** 10.3390/jof8050512

**Published:** 2022-05-16

**Authors:** Rui Zhong, Daniel A. Bastías, Xingxu Zhang, Chunjie Li, Zhibiao Nan

**Affiliations:** 1 State Key Laboratory of Grassland Agro-Ecosystems, Key Laboratory of Grassland Livestock Industry Innovation, Ministry of Agriculture, Center for Grassland Microbiome, College of Pastoral Agriculture Science and Technology, Lanzhou University, Lanzhou 730020, China; ruizhong810@sina.com (R.Z.); chunjie@lzu.edu.cn (C.L.); 2 AgResearch Limited, Grasslands Research Centre, Palmerston North 4442, New Zealand; daniel.bastias@agresearch.co.nz

**Keywords:** drought tolerance, photosynthesis, biomass, *Achnatherum inebrians*, *Epichloë* endophyte, transcriptomes

## Abstract

*Achnatherum inebrians* (drunken horse grass, DHG) plants, a dominant grass species in the arid and semi-arid regions of northwest China, symbiotic with an *Epichloë* fungal endophyte, is well adapted to drought. However, little is known about how the presence of the foliar *Epichloë* endophyte enhances the tolerance of DHG to drought at the molecular level. This study explored the positive effects of the presence of the *Epichloë* endophyte on plant growth, biomass, and photosynthetic efficiency and processes of DHG under non-drought and two drought (moderate and severe) treatments, using RNA sequencing to compare transcriptomes. The transcriptome results showed that 32 selected unigenes involved in the photosynthesis processes within *Epichloë* symbiotic plants were differently expressed (DEGs) versus non-symbiotic plants. The majority of these selected DEGs were upregulated in *Epichloë* symbiotic plants versus non-symbiotic plants, such as upregulated unigenes (c51525.graph_c1, c47798.graph_c0 & c64087.graph_c0) under drought conditions. In line with the transcriptomes data, the presence of the *Epichloë* endophyte promoted the photosynthetic rate and biomass accumulation of DHG plants, and the relationship between the photosynthetic rate and biomass is linear and significant. The presence of the endophyte only increased the biomass per tiller of DHG plants under drought. This study provides further insights into the molecular mechanisms that underlie the enhanced plant growth and drought tolerance of *Epichloë*-symbiotic DHG plants.

## 1. Introduction

Drought, a primary environmental factor, limits plant productivity in natural ecosystems [1]. In grasslands in the arid and semi-arid regions of northwest China, grasses, including forage species in the family Poaceae, are typically dominant species. Grassland species adapt and respond to drought through many strategies, such as changes in plant physical responses, biomass accumulation and/or allocation, and the accumulation of some protective metabolites [2,3]. Natural selection and plant breeding can also enhance drought tolerance [4]. Some symbiotic beneficial microbes (e.g., root arbuscular mycorrhizal fungi and foliar *Epichloë* endophytes) enhanced plant drought tolerance through many strategies, such as absorbing water and nutrients through external hyphae of mycorrhizal and ectomycorrhizal fungi under drought conditions [3,5].

About 20–30% of grass species in the family Poaceae may be symbiotic with a foliar *Epichloë* fungal endophyte, promoting plant growth and enhancing persistence [6,7,8]. *Epichloë* endophytes colonize the aboveground tissues of grasses but not the roots. Associations are generally symptomless, and transmission in many associations is entirely vertical in nature, through the seed of host plants [9]. The presence of an *Epichloë* sp. can modify and enhance growth to avoid drought damage [6,10,11,12]. Further, the presence of the *Epichloë* endophyte produces or induces the production of some bioactive metabolites (fungal alkaloids and phytohormone) to help the host plant to adapt to drought [6,11,13,14]. The presence of an *Epichloë* sp. also regulates the plant’s physical responses to reduce/eliminate plant damage from water deficiency, such as through stomata regulation, osmotic adjustment, and enhanced water use efficiency (WUE) [11,15,16]. Models predict that crop yield can be improved through enhancing efficiency of the photosynthetic process, and photosynthetic efficiency can be used to predict plant drought tolerance [17]. *Epichloë* symbiotic plants have been reported to have higher net photosynthetic efficiency and biomass accumulation than non-symbiotic plants [18,19,20], and this knowledge led us to assess if this enhancing effect was linked to drought tolerance. We assessed effects of the presence of an *Epichloë* endophyte in host grasses on the net photosynthetic rate through a standard meta-analysis based on published procedures.

*Achnatherum inebrians* (drunken horse grass, DHG) plants symbiotic with either *Epichloë gansuensis* or *E. inebrians,* are widely distributed in the arid and semi-arid regions of China, and the *Epichloë* infection rate of wild DHG plant populations could reach 100% [21,22,23]. DHG plants have the potential to be utilized as an animal feed because of high levels of crude proteins (close to that of *Medicago sativa*), in addition to the fact that DHG plants without the *Epichloë* endophyte do not cause disorders in grazing livestock [24]. *E. gansuensis* symbiotic DHG plants (EI) exposed to drought had greater plant biomass/nutrient accumulation and faster photosynthetic efficiency than those DHG plants without the endophyte (EF) in controlled pot experiments and in field trials in semi-arid regions [16,25,26]. Additionally, *Epichloë* symbiotic DHG plants had important ecological roles in plant competition and pathogen control in controlled conditions and natural grassland ecosystems [27,28,29]. Here, we studied the tolerance of *Epichloë*-associated DHG plants against drought stress. We subjected *E. gansuensis* symbiotic and non-symbiotic DHG plants to water restriction treatments (i.e., severe, moderate, and no drought). We hypothesized that plants associated with the *E. gansuensis* may exhibit high tolerance to the drought due to the *Epichloë*-mediated enhancement of the host plant photosynthesis. For this undertaking, we measured plant growth variables (as proxies of tolerance to the stress), and the photosynthetic capacity in EI and EF plants. In addition, we identified the specific photosynthesis-related genes linked to the variations in photosynthesis rates.

## 2. Material and Methods

### 2.1. Seed Origin, Plant Materials, and Experimental Design

Seeds of *E. gansuensis* symbiotic DHG plants were generated from one grass population collected from the grassland in Sunan County, Gansu, China (101°01′ E, 38°35′ N, attitude 3297 m). *Epichloë* non-symbiotic DHG plants were generated by treating symbiotic seeds from F0 generation with a systemic fungicide (Thiophanate-methyl, 70% effective component) with 100 times dilution and 2 h treatment [30]. In order to multiply seeds, fungicide-treated and untreated seeds were planted in contiguous plots in an experimental field of the Yuzhong campus of Lanzhou University. All DHG plants were checked via microscopic examination for the presence of seldom-branched hyphae characteristic of *Epichloë* spp. in leaf sheath pieces stained with aniline blue, in the seeds of individual plants, and plants were individually labeled as EI or EF plants, respectively. Additionally, we did not observe the effects of fungicide treatment on the morphology, phenology, and growth of our experimental plants through pot and field experiments [25,30,31]. Seeds were collected from EI and EF DHG plants grown in the experimental field and stored at −4 °C before planting.

Three EI or EF seeds were planted in one plastic pot (diameter: 24 cm, height: 15 cm) filled with 200 ± 2 g of sterilized vermiculite (120 °C for 5 h), and later thinned to one seedling per pot. Hoagland’s solution was used to quantitatively water these experimental pots every other day after the appearance of the second fully expanded leaf of individual plants [26]. Pots were maintained at a constant-temperature (26 ± 2 °C) greenhouse. After one month, pots containing similar sized EI (*n* = 27) and EF (*n* = 27) seedlings were cut 15 cm above the vermiculite surface, and the water-holding capacity of each pot was reduced to 15% relative saturation moisture content (RSMC) [26]. Subsequently, severe drought (SD, 15% RSMC), moderate drought (MD, 30% RSMC), and no drought (CK, 60% RSMC) treatments were established and sustained for 50 days. There were 9 replicates per treatment.

### 2.2. Differentially Expressed Genes

At the end of the soil moisture treatments, three fresh leaves of three EF or EF DHG plants for each soil moisture treatment were collected and immediately frozen in liquid nitrogen, and then stored at −80 °C for the subsequent RNA extraction and transcriptome sequencing (Supplementary Materials). Transcriptome analysis in the present study was performed by Biomarker Technologies (Beijing, China). A total amount of 3 μg RNA per sample was used as input material for the preparations of RNA samples. Sequencing libraries were generated using the NEBNext^®^Ultra™ RNA Library Prep Kit for Illumina^®^ (NEB, Ipswich, MA, USA). Afterwards, the libraries were sequenced by the Illumina HiSeq 2000 platform. The sequencing data used in this study have been deposited in Sequence Read Achieve (SRA) of the NCBI database under accession numbers PRJNA748183. The function of these unigenes was annotated based on the following databases on 8 May 2018:

NCBI Non-redundant protein sequences (NR, ftp://ftp.ncbi.nih.gov/blast/db/);

Protein family (Pfam, http://pfam.xfam.org/);

Clusters of Protein Homology (KOG, http://www.ncbi.nlm.nih.gov/KOG/);

Clusters of Orthologous Groups of proteins (COG, http://www.ncbi.nlm.nih.gov/COG/);

Orthologous groups of genes (eggNOG, http://eggnogdb.embl.de/);

A manually annotated and reviewed protein sequence (Swiss-Prot, http://www.uniprot.org/);

Kyoto Encyclopedia of Genes and Genomes (KEGG, http://www.genome.jp/kegg/);

Gene Ontology (GO, http://www.geneontology.org/).

Gene expression levels were estimated on the basis of fragments per kilobase of transcript per million mapped fragments (FPKM) by RSEM for each sample [32]. Clean data were mapped back onto the assembled transcriptome; read count for each gene was obtained from the mapping results. For each treatment, with three biological replicates, differential expression analysis of the two groups (the EF plants were the control group) was performed using the DESeq2 package in R software (version 1.10.1), which provides statistical routines for determining differential expression of genes using a model based on the negative binomial distribution. The resulting *p* values were adjusted using the Benjamini and Hochberg’s approach for controlling the false discovery rate (FDR) at 0.05. These log_2_ [fold changes (FC)] of unigene FPKM were used to identify whether these unigenes were differentially expressed genes (DEGs) [33]. We used the KOBAS2.0 software to test the statistical enrichment of DEGs in KEGG pathways [34,35]. DEGs involved in photosynthesis (ko00195) and photosynthesis antenna proteins (ko00196) from the present transcriptomic data were selected for further analysis. Here, a total of 32 unigenes were selected for the subsequent analysis.

Amino acid sequences of these selected unigenes were blasted (blastx) against the genome of a related species to get the referenced sequences, and the resulting and reference sequences were used to construct Neighbor-Joining (NJ) phylogenetic trees using Molecular Evolutionary Genetics Analysis (MEGA, version 10.0.5) software [36]. Total leaf RNA of each sample used for RNA-Seq analysis was also used for quantitative real-time PCR (qRT-PCR) analysis. Single-stranded cDNAs were synthesized from 2.5 µg of total RNA with MMLV reverse transcriptase (TaKaRa, Dalian, China). The qRT-PCR was performed using SYBR Premix Ex Taq II Kit (TaKaRa, Dalian, China) on a 7500 Fast Real-time PCR system (Applied Biosystems, Waltham, MA, USA). The specific primers sequences of the selected unigenes used in the present study are listed in Table S1. Three technical replicates were carried out for each reaction, and the relative expression levels were normalized to the expression of the unigene (ID: c56016.graph_c0 detected in the present study) and calculated using the 2^−^^△△CT^ method.

### 2.3. Indexes of Plant Growth and Photosynthesis

In order to test the tolerance of *Epichloë* symbiotic DHG plants to severe and moderate drought, we assessed the indices of plant growth (plant height, tiller number and biomass), chlorophyll content, and photosynthesis (photosynthetic rate, intercellular carbon dioxide (CO_2_) concentration, stomatal conductance, and transpiration rate). The heights and tiller numbers of EI and EF DHG plants were measured upon completion of the soil moisture treatments. The effects of the presence of the *Epichloë* endophyte on the photosynthetic rate of host plants were obtained through a standard meta-analysis (see the detailed information in the Supplementary Materials).

The chlorophyll content of three leaves of one individual EI or EF DHG plant was measured using a chlorophyll meter (SPAD-502Plus, Konica Minolta Sensing, Inc., Osaka, Japan). Then, the mean of three measurements represented the actual value of this individual plant. The photosynthetic indexes were performed using a portable photosynthesis system (LI-6400, LI-COR, Lincoln, NE, USA) between 9:00 and 11:00 on the morning of the final day of soil water treatments. The concentration of air CO_2_ was 410 ± 10 μmol CO_2_ mol^−1^, the chamber was equipped with a red/blue LED light source (LI6400-02B), with the photo flux density set at 1200 μmol m^−2^s^−1^, and the detection conditions were at 28 ± 1 °C. Finally, the shoots and roots of sampled plants were collected from the nine pots to measure the fresh weight of shoots and roots, and then the dry weight of shoots and roots were recorded when a constant weight had been reached in an 80 °C oven for 48 h.

### 2.4. Data Analysis of Plant Parameters

Differences in plant growth performance, biomass, and photosynthetic index under the *Epichloë* endophytic status and different soil moisture levels were tested using a two-way analysis of variance (ANOVA) using the datarium package of R software. A statistically significant two-way interaction was followed up by simple main effect analyses; that is, evaluating the effect of endophytic status during each soil moisture treatment. All values are means ± SE of the mean.

## 3. Results

### 3.1. Differentially Expressed Genes in Photosynthesis

**Figure 1:**
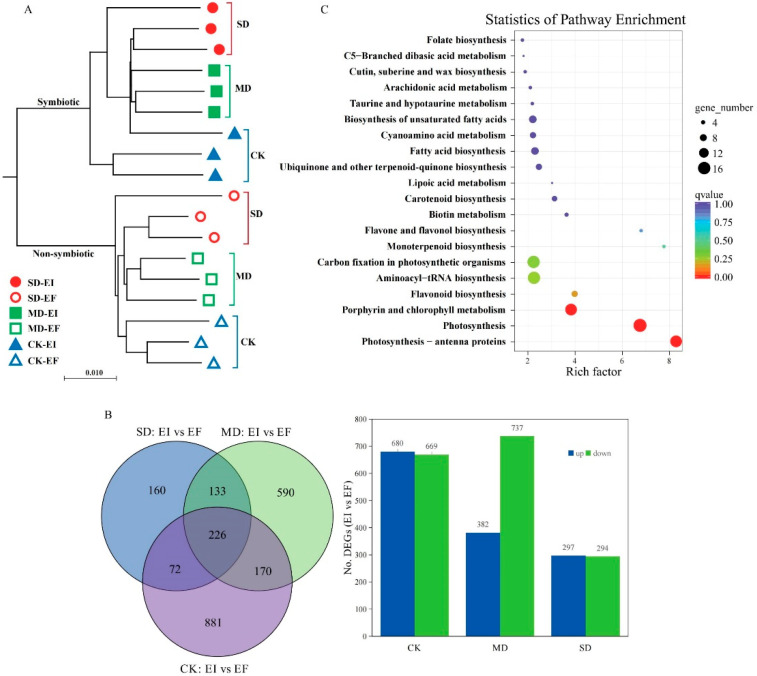
The unigenes and differently expressed genes (DEGs) based on the leaf transcriptome data of *Achnatherum inebrians* plants in response to the presence of the *Epichloë* fungal endophyte under non-drought (CK), moderate (MD), and severe (SD) drought treatments. Note: (**A**): The sample similarity analysis of all unigenes detected in the leaves of *Epichloë* symbiotic (EI) and non-symbiotic (EF) plants under CK, MD, and SD moisture treatments. (**B**): The number of all and up/down-regulated DEGs in EI plants versus EF plants under CK, MD, and SD moisture treatments. (**C**): Kyoto Encyclopedia of genes and genomes (KEGG) pathway enrichment of DEGs in response to the *Epichloë* endophyte under CK treatment; the dot color represents the enrichment q-value of the related pathway, and the dot scale indicates the number of DEGs in the pathway.

A total of 462,911,295 clean reads were obtained from all samples, and 64.88–70.95% reads of each sample were mapped and used for further analysis (Table S2). A total of 92,964 unigenes were detected from 18 samples with the mean length for all unigenes being 865.18 bp, with an N50 length of 1677 bp (Table S3). A total of 42,618 (45.85%) unigenes were annotated in eight different public databases, including COG (13.27%), GO (27.22%), KEGG (13.40%), KOG (24.20%), Pfam (29.05%), Swissprot (22.20%), eggnog (41.95%), and Nr (39.62%) databases (Table S4). The results indicated that expression of all unigenes differed between EI and EF plants regardless of non-drought and drought treatments (Figure 1A and Figure S1). Few DEGs in DHG plants in the drought (SD: 116 DEGs and MD: 11 DEGs) treatments versus CK treatment were detected (Figure S2). There were 1349 (680 up and 669 down), 1119 (382 up and 737 down), and 591 (297 up and 294 down) DEGs in EI DHG plants versus EF plants under CK, MD, and SD treatments, respectively (Figure 1B).

KEGG pathways (top 20) results indicated that these DEGs involved in the photosynthesis processes responded to the presence of the *Epichloë* endophyte in the CK treatment, such as for photosynthesis (ko00195), antenna proteins (ko00196), chlorophyll metabolism, carbon fixation in photosynthetic organisms, and other metabolites processes (Figure 1C). Similar unigenes of these DEGs are also reported in some crop and model plants in the sub-family Pooideae. The identity of these DEGs with reference genes was over 80%, from 80.18% to 100% (Table 1). There were 16 DEGs associated with the process of photosynthesis, such as photosystem II (8 DEGs: *psbB*, *psbC*, *psbE*, two *psbS*, *psbQ,* and *psb27*), photosystem I (3 DEGs: *psaO*, *psaE,* and *psaG*), photosynthetic electron transport (5 DEGs: *petE*, *petF*, two *petH,* and *petJ*), and F-type ATPase (1 DEG: *atpH*) (Table 1 and Figure S3). There were 16 DEGs identified that were associated with the process of photosynthesis antenna proteins, including *lhcB1* (10), *lhcB2* (2), *lhcB3* (1) and *lhcB5* (2), and *lhcB6* (1) unigenes (Table 1 and Figure S3).

**Figure 2:**
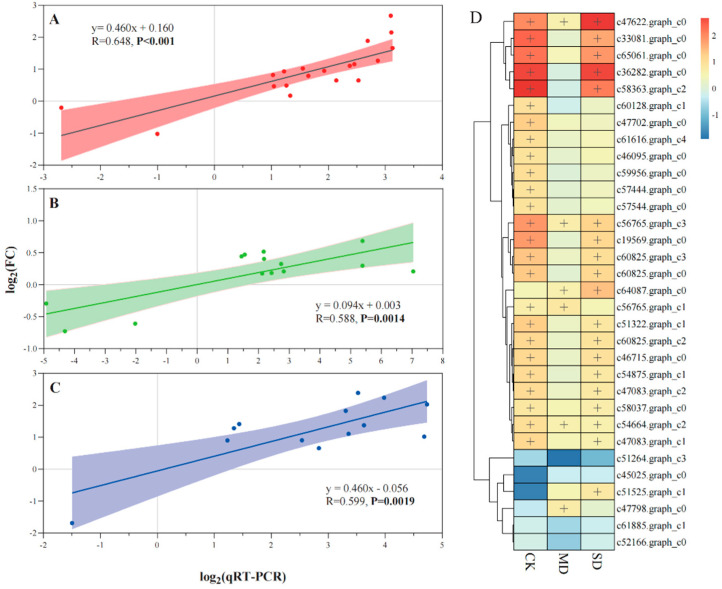
Validation of the expression changes (log_2_ (fold change, FC)) and a heatmap (FC > 1.5, plus) of selected genes involved in the processes of photosynthesis and photosynthesis-antenna proteins from RNA-Seq using qRT-PCR under normal (CK, (**A**)), moderate drought (MD, (**B**)), and severe drought (SD, (**C**)) moisture treatments. Note: The results are plotted for genes that show the significant upregulation and down regulation of leaves in response to the fungal endophyte under different soil moisture levels (**D**); a plus indicates the upregulated DEGs in *Epichloë* symbiotic (EI) versus non-symbiotic (EF) plants under CK, MD, and SD moisture treatments. The linear trend line, the R-value, and 95% confidence interval are shown.

Significant positive linear relationships between transcriptome data and qRT-PCR were observed in CK (R = 0.648, *p* < 0.001), MD (R = 0.588, *p* < 0.01) and SD (R = 0.599, *p* < 0.01) treatments, respectively (Figure 2A–C). The majority of selected DEGs in photosynthesis and antenna proteins were upregulated in EI DHG plants versus EF DHG plants under CK, MD, and SD treatments, respectively (Figure 2D). DEGs (e.g., c51525.graph_c1, c47798.graph_c0 & c64087.graph_c0) were only upregulated in the EI versus EF DHG plants under drought conditions (Figure 2B–D). *Epichloë* presence upregulated three DEGs (c47622.graph_c0, c56765.graph_c3 & c54664.graph_c2) expression regardless of non-drought and drought treatments (Figure 2). Meanwhile, the majority of DEGs were upregulated in CK and SD treatments (Figure 2A,C,D).

### 3.2. Photosynthesis

**Figure 3:**
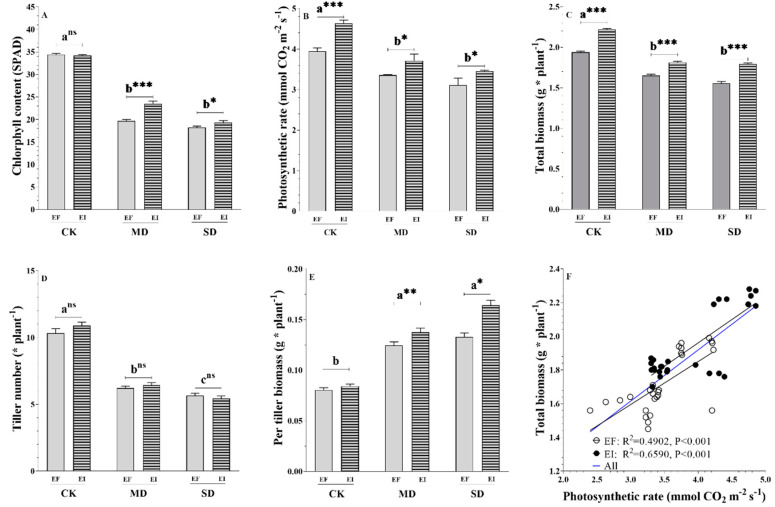
The chlorophyll content (**A**), photosynthetic rate (**B**), total biomass (**C**), the tiller number (**D**), and per tiller biomass (**E**) of *Epichloë* symbiotic (EI) and non-symbiotic (EF) plants under normal (CK), moderate drought (MD), and severe drought (SD) treatments, and liner linear regression (**F**) between photosynthetic rate and total biomass. Note: Different lowercase letters mean significant difference at *p* < 0.05 among three soil moisture treatments at the 0.05 level. The *, ** and *** mean significant differences between EI and EF plants at the corresponding water content at the 0.05, 0.01 and 0.001 levels, respectively.

As the consequence of the upregulation of the majority of photosynthesis DEGs, we assessed whether photosynthetic rates were higher in EI versus EF DHG plants. Two-way ANOVA results indicated that plant chlorophyll content and photosynthetic indices responded differently to the drought treatments and the *Epichloë* presence (Table 2 and Table S5). The effects of the presence of the *Epichloë* endophyte on leaf chlorophyll content depended on the soil moisture and symbiosis x soil moisture treatments: F _(2,48)_ = 15.73, *p* = 0.000 (Table 2). The *Epichloë* presence significantly increased the leaf chlorophyll content only in the MD and SD treatments in 19.7% (*p* = 0.000) and 7.1% (*p* = 0.040), respectively (Figure 3A). The chlorophyll content in EI DHG plants was only significantly higher than in EF DHG plants under MD and SD treatments (Figure 3A). The MD and SD treatments reduced the photosynthetic efficiency of DHG plants compared to the CK level of soil moisture: F_(2,48)_ = 47.80, *p* < 0.001 (Figure 3B). *Epichloë* presence increased the photosynthetic efficiency of DHG plants regardless of soil moisture treatments, symbiosis status: F_(1, 48)_ = 14.08, *p* < 0.001, with increases of 17.2% (*p* = 0.000), 10.7% (*p* = 0.022) and 10.9% (*p* = 0.030) under CK, MD and SD treatments, respectively (Figure 3B). The intercellular carbon dioxide concentration was significantly higher in EI versus EF DHG plants under two drought conditions, with an increase of 19% and 22% under MD and SD, respectively (Figure S4A). The transpiration rate was only significantly lower in EI versus EF plants under non-drought conditions (Figure S4C).

### 3.3. Plant Growth and Biomass

The plant performance and shoot/root/total biomass significantly responded to the presence of the *Epichloë* endophyte and drought treatments (Table 1 and Table S5). The effect of *Epichloë* on plant height depended on the soil moisture, symbiosis x soil moisture treatments: F _(2,48)_ = 17.27, *p* = 0.000 (Table S5). The *Epichloë* endophyte significantly increased the plant height only in the MD and SD treatments by 13.1% (*p* = 0.000) and 9.6% (*p* = 0.000), respectively (Figure S1A). The effects of the *Epichloë* endophyte on total biomass depended on the soil moisture, symbiosis x soil moisture treatments: F _(2,48)_ = 7.67, *p* = 0.001 (Table S5). The *Epichloë* endophyte significantly increased the total biomass in the CK, MD, and SD treatments in 12.7% (F _(1,16)_ = 288.0, *p* = 0.000), 11.3% (F_(1,16)_ = 58.9, *p* = 0.000) & 21.4% (F_(1,16)_ = 88.1, *p* = 0.000), respectively (Figure 3C). The drought treatments significantly decreased the tiller number of DHG plants compared with CK treatments regardless of symbiosis status, F _(2,48)_ = 17.27, *p* = 0.000 (Figure 3D). Here, the present results indicated that the *Epichloë* endophyte only had significant positive effects on the average per-tiller biomass under the MD and SD treatments (Figure 3E). Additionally, the total biomass of EI and EF DHG plants was significantly (*p* < 0.001) and positively associated with the photosynthetic efficiency regardless of the presence or absence of the *Epichloë* endophyte (Figure 3F).

### 3.4. Meta-Analysis

**Figure 4:**
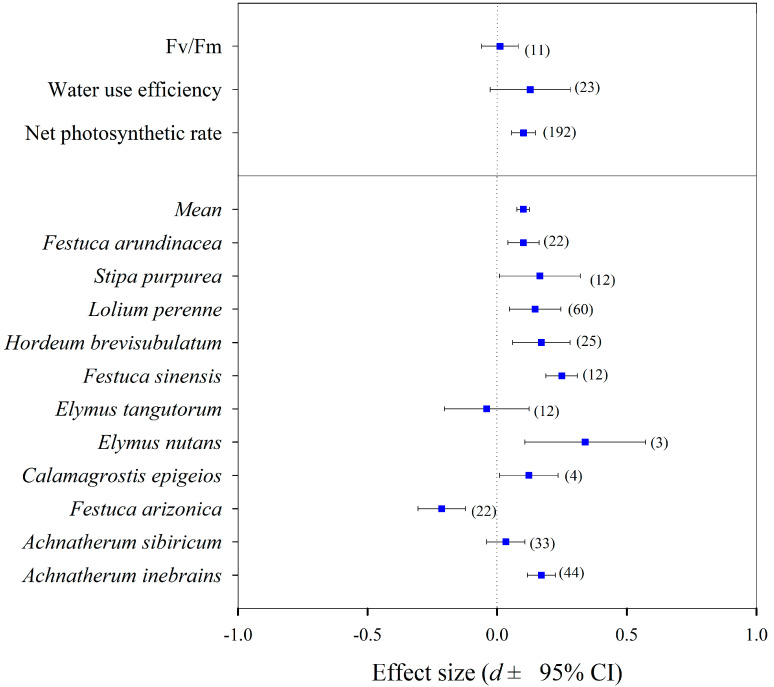
The effects of the symbiotic *Epichloë* fungal endophyte on the net photosynthetic efficiency (NPE), water use efficiency (WUE), and photochemical efficiency (Fv/Fm) of grasses (relative effects of *Epichloë* symbiotic versus non-symbiotic plants). Note: A total of 45 papers, including 14 papers in English and 31 papers in Chinese, were selected for this analysis. The number of studies analysed in each category are given in parentheses.

There were more positive effects of the *Epichloë* endophyte on the net photosynthetic efficiency (NPE) (effect size = 0.102, 95% CI = 0.057 to 0.148), the water use efficiency (effect size = 0.128, 95% CI = −0.027 to 0.283), and the photochemical efficiency (effect size = 0.011, 95% CI = −0.060 to 0.082) of the EI plants than the EF plants through the present meta-analysis (Figure 4). There was an overall positive effect (main effect size = 0.101, 95% CI = 0.077 to 0.125) of the *Epichloë* endophyte on NPE of the EI plants compared to the EF plants (Q_b_ = 98.2, *p* = 0.000, df = 10) (Figure 4). The higher NPE were found in DHG (effect size = 0.170, 95% CI = 0.115 to 0.226), *A. sibiricum* (effect size = 0.034, 95% CI = 0.032 to 0.099), *Calamagrostis epigeios* (effect size = 0.122, 95% CI = −0.207 to 0.451), *Festuca sinensis* (effect size = 0.249, 95% CI = 0.132 to 0.367), *Hordeum brevisubulatum* (effect size = 0.171, 95% CI = 0.095 to 0.246), *Lolium perenne* (effect size = 0.147, 95% CI = 0.094 to 0.200), *Stipa purpurea* (effect size = 0.165 95% CI = 0.039 to 0.291), and *F. arundinacea* (effect size = 0.102, 95% CI = 0.015 to 0.189) plants infected by *Epichloë* endophyte (Figure 4), while the lower NPE were only observed in the *F. arizonica* (effect size = 0.214, 95% CI = −0.297 to −0.131) and *Elymus tangutorum* (effect size = 0.040, 95% CI = −0.169 to 0.088) plants infected by the *Epichloë* endophyte (Figure 4).

## 4. Discussion

**Figure 5:**
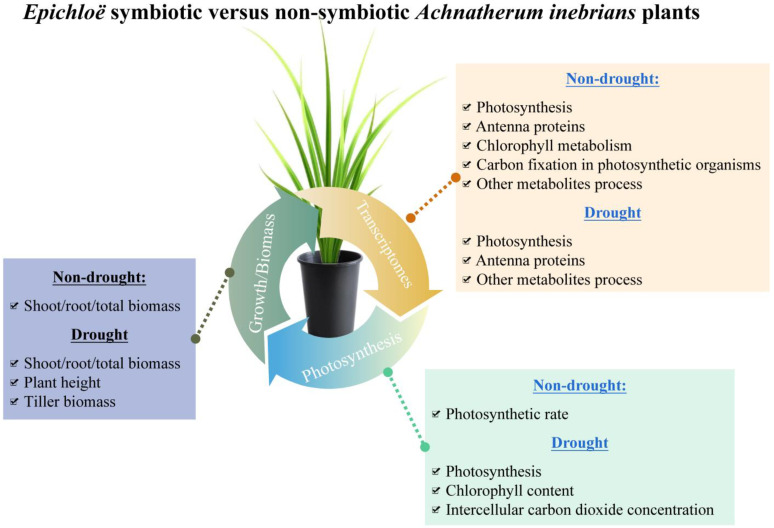
The overview diagram for understanding the advantages of *Epichloë* symbiotic *Achnatherum inebrians* plants versus non-symbiotic plants combining the transcriptomes, photosynthesis, and growth.

With plants exposed to a water deficit, production was linked to the net photosynthetic efficiency. Adaption mechanisms of plants responding to abiotic and biotic stresses include the forming of symbiotic associations with beneficial microbes. In this study, which we conducted to examine the effects of different levels of drought stress on *A. inebrians* plants with and without an *Epichloë* systemic endophyte, our results provide a comprehensive overview of unigene changes associated with photosynthesis processes (Figure 5). We found that the majority of DEGs in photosynthesis were upregulated in *Epichloë* symbiotic plants and thus had a higher NPE than non-symbiotic plants (Figure 5).

Many studies have confirmed that the presence of an *Epichloë* endophyte promoted plant growth, biotic resistance, and abiotic tolerance of their host grasses [3,8,36,37,38]. Studies have provided an understanding of how symbiotic microbes improved drought tolerance through different strategies [5,11,39,40]. The secondary metabolites produced or induced by the presence of an *Epichloë* endophyte contribute to a plant’s abiotic/biotic tolerance [38,39]. Plant metabolite processes begin with the products of the photosynthesis process. The presence of an *Epichloë* endophyte in grasses stimulates the accumulation of plant metabolites in the aboveground tissues and in roots, such as SA, flavonoids, and total phenolic compounds [39,41,42,43]. As we expected, the unigenes in flavonoids and fatty acids biosynthesis were differently expressed in response to the *Epichloë* endophyte.

The presence of *Epichloë* spp. in aboveground tissues altered the transcription levels of their host cool-season grasses [29,44,45,46,47]. The expression of dehydrin and heat shock protein genes in *F. arundinacea* was enhanced by the symbiotic *Epichloë* endophyte in water-unstressed conditions [47]. Similarly, the majority of unigenes that differently responded to the *Epichloë* presence were found in the control, and in abiotic and biotic conditions such as those involved with SA biosynthesis [14,29]. As expected, this study also detected a large number of DEGs in EI plants versus EF DHG plants under the three soil moisture treatments, and the DEGs detected in the present transcriptome data included some involved in photosynthesis (PSI, PSII, and PET), which are in line with our hypothesis. Photosynthesis begins with harvesting light within leaves, and the present transcriptome data indicated that genes in antenna proteins and the chlorophyll metabolism process were upregulated in response to the *Epichloë* endophyte, and a higher chlorophyll content was found in EI versus EF DHG plants. The present results are also supported by two studies [19,48]. The abundance of lhcI and lhcII proteins in EI *D. glomerata* plants was higher than that of EF plants [19]. Meanwhile, the genes of lhcI type II were upregulated in the *E. festucae*-infected *F. rubra* compared to EF plants [48].

Ambrose and Belanger (2012) also noted that some genes involved in the photosynthesis process of red fescue (*F. rubra*) are upregulated and downregulated in response to the presence of an *Epichloë* endophyte [48]. The *Epichloë* endophyte increased the rate of carbon assimilation, PSII photochemistry, and grass biomass associated with *D. glomerate* plants [19]. A study showed that the 1000 D7 gene (CP47) was downregulated in perennial ryegrass symbiotic with *E. festucae* var. *lolii* [44]. Similar to the study of *Epichloë* symbiotic perennial ryegrass, our results indicated that a unigene (c61885.graph_c1, encoding PSII CP47 reaction center protein) was downregulated in the EI DHG plants regardless of non-drought and drought treatments. Another study found that the electron transport rate was enhanced by 31% in EI plants and reduced by 13% in EF plants under water stressed versus CK treatments [49]. As we expected, one (c51525.graph_c1, *petH*) and two (c47702.graph_c0, *petF*; c46095.graph_c0, *petJ*) upregulated unigenes were only in EI DHG plants with under drought and non-drought treatments, respectively. This is in line with a study that indicated that the NADPH activity in EI *D. glomerata* plants was significantly greater (c. 4.28) than in EF plants [19]. Our results and present meta-analysis indicated that the *Epichloë* endophyte promoted the photosynthetic rate of host plants (Figure 5). Meanwhile, the presence of the *Epichloë* endophyte on the net photosynthesis rate of *L. perenne* is independent of endophyte concentration *in planta* [18].

Greater photosynthetic rates are commonly associated with higher production, and our results showed the liner relationship between photosynthetic rates and the total biomass of EI and/or EF DHG plants. In line with our hypothesis and certain studies, the presence of an *Epichloë* endophyte increased the biomass and photosynthetic rate of host plants under drought [12,16,25,26]. Another study found that EI *F. arizonica* plants produced more shoot biomass and had a greater plant growth rate versus EF plants under low water availability [10]. Our results have shown that the presence of the *Epichloë* endophyte had no effect on the per-tiller biomass under non-drought treatment, while it did, however, promote the per-tiller biomass accumulation under (MD and SD) drought conditions. This study provides an enhanced understanding of the enhancement of drought tolerance in *Epichloë* symbiotic plants (Figure 5).

## Figures and Tables

**Table 1:** Selected unigenes associated with the processes of photosynthesis antenna proteins and photosynthesis in *Achnatherum inebrians* identified in the RNA-seq analysis, including photosystem II (PSII), photosystem I (PSI), light-harvesting complex II, and chlorophyll a/b binding protein (lhcB).

Unigene ID	Description	Top Blast	Identity
c61885.graph_c1	PSII CP47 reaction center protein	KM974729.1	99.57%
c59956.graph_c0	PSII CP43 chlorophyll apoprotein	YP_009156694.1	100.00%
c51264.graph_c3	PSII cytochrome b559 subunit alpha	MK593558.1	82.80%
c47798.graph_c0	PSII 22kDa protein	WP_119617769.1	80.18%
c57444.graph_c0	PSI subunit PsaO	BAJ90241.1	92.65%
c60128.graph_c1	PSII oxygen-evolving enhancer protein 2	KAF0929002.1	87.78%
c54875.graph_c1	PSII oxygen-evolving enhancer protein 2	XM_003557926.4	86.96%
c58037.graph_c0	PSII oxygen-evolving enhancer protein 3	ABG75753.1	94.33%
c51322.graph_c1	PSII Psb27 protein	XP_003563195.1	88.51%
c47702.graph_c0	PSI subunit IV	XM_003559195.4	83.87%
c61616.graph_c4	cytochrome c6	WP_149346037.1	97.69%
c52166.graph_c0	plastocyanin	XM_020303247.1	84.16%
c47702.graph_c0	ferredoxin	P00228.2	89.58%
c45025.graph_c0	ferredoxin	XP_003558196.1	92.33%
c51525.graph_c1	ferredoxin--NADP+ reductase	RLM86482.1	91.06%
c46095.graph_c0	ferredoxin--NADP+ reductase	XP_020192565.1	93.15%
c57544.graph_c0	cytochrome c6	XM_020322066.1	88.89%
c19569.graph_c0	F-type H^+^-transporting ATPase subunit delta	OAY75305.1	90.67%
c33081.graph_c0	lhcB protein 1	KAE8804052.1	88.41%
c36282.graph_c0	lhcB protein 1	AK450185.1	80.54%
c47622.graph_c0	lhcB protein 1	PNX82175.1	98.04%
c56765.graph_c1	lhcB protein 1	XP_010237260.1	92.68%
c56765.graph_c3	lhcB protein 1	JF747382.1	97.22%
c58363.graph_c2	lhcB protein 1	XM_010238958.3	81.22%
c60825.graph_c3	lhcB protein 1	XM_020308118.1	85.50%
c64087.graph_c0	lhcB protein 1	VAH08634.1	100.00%
c65061.graph_c0	lhcB protein 1	AK446357.1	81.93%
c60825.graph_c0	lhcB protein 1	XP_003562323.1	98.62%
c60825.graph_c2	lhcB protein 2	1707316B	97.47%
c54664.graph_c2	lhcB protein 2	XP_003562898.1	96.49%
c47083.graph_c1	lhcB protein 3	XP_003577654.1	100.00%
c47083.graph_c2	lhcB protein 5	XP_006662849.1	83.00%
c46715.graph_c0	lhcB protein 5	XP_020200697.1	95.06%

**Table 2:** ANOVA table showing the effects of plant symbiosis status and soil moisture levels on the tiller number, biomass, chlorophyll content, and photosynthetic rate of *Achnatherum inebrians* plants symbiotic with the fungal endophyte *Epichloë gansuensis*.

Response Variable	Treatments	*Df*(*n*,*d*)	F	*p*-Value
Chlorophyll content (*n* = 9)	Symbiosis	1,48	18.128	**<0.001**
	Soil moisture	2,48	127.886	**<0.001**
	Symbiosis x Soil moisture	2,48	8.925	**<0.001**
Photosynthetic rate(mmol CO_2_ m^−^^2^ s^−1^) (*n* = 9)	Symbiosis	1,48	27.700	**<0.001**
	Soil moisture	2,48	34.208	**<0.001**
	Symbiosis x Soil moisture	2,48	1.962	0.152
Tiller number (#*plant^−1^) (*n* = 9)	Symbiosis	1,48	1.064	0.308
	Soil moisture	2,48	306.511	**<0.001**
	Symbiosis x Soil moisture	2,48	1.574	0.218
Total biomass (g) (*n* = 9)	Symbiosis	1,48	339.720	**<0.001**
	Soil moisture	2,48	424.020	**<0.001**
	Symbiosis x Soil moisture	2,48	7.670	**0.001**
per-tiller biomass (g) (*n* = 9)	Symbiosis	1,48	29.740	**<0.001**
	Soil moisture	2,48	183.900	**<0.001**
	Symbiosis x Soil moisture	2,48	7.850	0.001

Note: Statistically significant effects are highlighted in bold.

## Data Availability

All data supporting the findings of this study are available within the paper and within its supplementary materials published online.

## Supplementary Materials

The following supporting information can be downloaded at: https://www.mdpi.com/article/10.3390/jof8050512/s1, Figure S1: Principal component analysis (PCA) of all unigenes from *Epichloë* symbiotic (EI) and non-symbiotic (EF) Achnatherum inebrians plants under normal (CK), moderate (MD) and severe (SD) drought treatments. Figure S2: The number of up- and down-regulated differently expressed unigenes (DEGs, FC > 1.5) of *Achnatherum inebrians* plants under the several (SD) and moderate drought (MD) treatments compared to the normal (CK) treatment (DEGs in endophyte-infected plants versus endophyte-free plants). Figure S3: The Neighbor-Joining (NJ) tree showing the amino acid sequences of differently expressed genes (DEGs) associated with photosynthesis (A) and photosynthesis-antenna proteins (B,C) of *Achnatherum inebrians* plants. All bootstrap values > 70% are shown (1000 replicates). Numbers above branches indicate the bootstrap values of the maximum likelihood analysis. Figure S4: The intercellular carbon dioxide concentration (A), transpiration rate (B) and stomatal conductance (C) of *Epichloë* symbiotic (EI) and non-symbiotic (EF) *Achnatherum inebrians* plants under normal (CK), moderate (MD) and severe (SD) drought treatments. The asterisk (*) means significant difference at *p* < 0.05 (independent *t*-test) between and EI and EF plants at corresponding water content at 0.05 level. The A and B mean significant differences among corresponding water content at 0.05 level. Figure S5: The fresh weight of shoot (a) and root (b), and dry weight of shoot (c) and root (d) of *Epichloë* symbiotic (EI) and non-symbiotic (EF) *Achnatherum inebrians* plants under normal (CK), moderate (MD) and severe (SD) drought treatments. The asterisk (*) means significant difference at *p* < 0.05 (independent *t*-test) between and EI and EF plants at corresponding water content at 0.05 level. The A and B mean significant differences among corresponding water content at 0.05 levels. Table S1: Selected unigenes associated with processes of photosynthesis and photosynthesis-antenna proteins identified in the RNA-seq analysis in the present study. Table S2: The comparative statistics between RNA sequencing clean data and transcriptome assembly of *Epichloë* symbiotic (EI) and non-symbiotic (EF) *Achnatherum inebrians* plants under normal (CK), moderate (MD) and severe (SD) drought treatments. Table S3: Length distribution of transcripts and unigenes of *Achnatherum inebrians* plants. Table S4: Unigenes statistics of *Achnatherum inebrians* plants transcriptome against eight different public databases. Table S5: The ANOVA table showing the effects of symbiosis status and soil moisture levels on the growth partners, biomass, chlorophyll content and photosynthetic indices of *Achnatherum inebrians* plants.

## References

[1] Ciais P., Reichstein M., Viovy N., Granier A., Ogee J., Allard V., Aubinet M., Buchmann N., Bernhofer C., Carrara A.J.N. (2005). Europe-wide reduction in primary productivity caused by the heat and drought in 2003. Nature.

[2] Cornelius C., Leingärtner A., Hoiss B., Krauss J., Steffan-Dewenter I., Menzel A. (2013). Phenological response of grassland species to manipulative snowmelt and drought along an altitudinal gradient. J. Exp. Bot..

[3] Hewitt K.G., Popay A.J., Hofmann R.W., Caradus J.R. (2021). *Epichloë*-a lifeline for temperate grasses under combined drought and insect pressure. Grass Res..

[4] Langridge P., Reynolds M. (2021). Breeding for drought and heat tolerance in wheat. Theor. Appl. Genet..

[5] De Vries F.T., Griffiths R.I., Knight C.G., Nicolitch O., Williams A. (2020). Harnessing rhizosphere microbiomes for drought-resilient crop production. Science.

[6] Decunta F.A., Pérez L.I., Malinowski D.P., Molina-Montenegro M.A., Gundel P.E. (2021). A systematic review on the effects of *Epichloë* fungal endophytes on drought tolerance in cool-season grasses. Front. Plant. Sci..

[7] Schardl C.L., Leuchtmann A., Spiering M.J. (2004). Symbioses of grasses with seedborne fungal endophytes. Ann. Rev. Plant Biol..

[8] Gibert A., Tozer W., Westoby M. (2019). Plant performance response to eight different types of symbiosis. New Phytol..

[9] Lee K., Missaoui A., Mahmud K., Presley H., Lonnee M. (2021). Interaction between grasses and *Epichloë* endophytes and its significance to biotic and abiotic stress tolerance and the rhizosphere. Microorganisms.

[10] Christensen M.J., Bennett R.J., Ansari H.A., Koga H., Johnson R.D., Bryan G.T., Simpson W.R., Koolaard J.P., Nickless E.M., Voisey C.R. (2008). *Epichloë* endophytes grow by intercalary hyphal extension in elongating grass leaves. Fungal. Genet. Biol..

[11] Morse L.J., Day T.A., Faeth S.H. (2002). Effect of *Neotyphodium* endophyte infection on growth and leaf gas exchange of Arizona fescue under contrasting water availability regimes. Environ. Exp. Bot..

[12] Xu W.B., Li M.M., Lin W.H., Nan Z.B., Tian P. (2021). Effects of *Epichloë sinensis* endophyte and host ecotype on physiology of *Festuca sinensis* under different soil moisture conditions. Plants.

[13] Nagabhyru P., Dinkins R.D., Wood C.L., Bacon C.W., Schardl C.L. (2013). Tall fescue endophyte effects on tolerance to water-deficit stress. BMC Plant Biol..

[14] Zhao Z.R., Kou M.Z., Zhong R., Xia C., Christensen M.J., Zhang X.X. (2021). Transcriptome analysis revealed plant hormone biosynthesis and response pathway modification by *Epichloë gansuensis* in *Achnatherum inebrians* under different soil moisture availability. J. Fungi.

[15] Elmi A.A., West C.P. (1995). Endophyte infection effects on stomatal conductance, osmotic adjustment and drought recovery of tall fescue. New Phytol..

[16] Xia C., Christensen M.J., Zhang X.X., Nan Z.B. (2018). Effect of *Epichloë gansuensis* endophyte and transgenerational effects on the water use efficiency, nutrient and biomass accumulation of *Achnatherum inebrians* under soil water deficit. Plant Soil.

[17] Ort D.R., Merchant S.S., Jean A., Alice B., Blankenship R.E., Ralph B., Roberta C., Hanson M.R., Hibberd J.M., Long S.P. (2015). Redesigning photosynthesis to sustainably meet global food and bioenergy demand. Proc. Natl. Acad. Sci. USA.

[18] Spiering M.J., Greer D.H., Schmid J. (2006). Effects of the fungal endophyte, *Neotyphodium lolii*, on net photosynthesis and growth rates of perennial ryegrass (*Lolium perenne*) are independent of in planta endophyte concentration. Ann. Bot..

[19] Rozpądek P., Wężowicz K., Nosek M., Ważny R., Tokarz K., Lembicz M., Miszalski Z., Turnau K. (2015). The fungal endophyte *Epichloë typhina*, improves photosynthesis efficiency of its host orchard grass (*dactylis glomerata*). Planta.

[20] Zhang Y.P., Zhou Y.F., Zhang X.X., Duan T.Y., Nan Z.B. (2018). Effects of *Epichloë* endophyte on antioxidant enzymes activities, photosynthesis and growth of three ecotypes of *Elymus dahuricus*. Front. Agric. Sci. Eng..

[21] Nan Z.B., Li C.J. Neotyphodium in native grasses in China and observations on endophyte/host interactions. Proceedings of the 4th International Neotyphodium/Grass Interactions Symposium.

[22] Li C.J., Nan Z.B., van Paul H., Dapprich P.D., Liu Y. (2004). A new *Neotyphodium* species symbiotic with drunken horse grass (*Achnatherum inebrians*) in China. Mycotaxon.

[23] Chen L., Li X.Z., Li C.J., Swoboda G.A., Young C.A., Sugawara K., Leuchtmann A., Schardl C.L. (2015). Two distinct *Epichloë* species symbiotic with *Achnatherum inebrians*, drunken horse grass. Mycologia.

[24] Liang Y., Wang H.C., Li C.J., Nan Z.B., Li F.D. (2017). Effects of feeding drunken horse grass infected with *Epichloë gansuensis* endophyte on animal performance, clinical symptoms and physiological parameters in sheep. BMC Vet. Res..

[25] Zhang X.X., Xia C., Nan Z.B. (2015). Effects of symbiotic *Epichloë gansuensis* endophyte on drunken horse grass (*Achnatherum inebrians*) growth and seed production. N. Z. J. Agric. Res..

[26] Xia C., Li N.N., Zhang X.X., Feng Y., Christensen M.J., Nan Z.B. (2016). An *Epichloë* endophyte improves photosynthetic ability and dry matter production of its host *Achnatherum inebrians* infected by *Blumeria graminis* under various soil water conditions. Fungal Ecol..

[27] Yao X., Fan Y.B., Chai Q., Johnson R.D., Nan Z.B., Li C.J. (2016). Modification of susceptible and toxic herbs on grassland disease. Sci. Rep..

[28] Yao X., Chen Z.J., Wei X.K., Chen S.H., White J., Huang X., Li C.J., Nan Z.B. (2020). A toxic grass *Achnatherum inebrians* serves as a diversity refuge for the soil fungal community in rangelands of northern China. Plant Soil.

[29] Kou M.Z., Bastías D.A., Christensen M.J., Zhong R., Nan Z.B., Zhang X.X. (2021). The plant salicylic acid signalling pathway regulates the infection of a biotrophic pathogen in grasses associated with an *Epichloë* endophyte. J. Fungi.

[30] Li N.N., Zhao Y.F., Xia C., Zhong R., Zhang X.X. (2016). Effects of thiophanate methyl on seed borne *Epichloë* fungal endophyte of *Achnatherum inebrians*. Pratac. Sci..

[31] Zhong R., Xia C., Ju Y., Zhang X.X., Duan T.Y., Nan Z.B., Li C.J. (2021). A foliar *Epichloë* endophyte and soil moisture modified belowground arbuscular mycorrhizal fungal biodiversity associated with *Achnatherum inebrians*. Plant Soil.

[32] Li B., Colin N.D. (2011). RSEM: Accurate transcript quantification from RNA Seq data with or without a reference genome. BMC Bioinform..

[33] Storey J.D. (2003). The positive false discovery rate: A Bayesian interpretation and the q-value. Ann. Stat..

[34] Mao X., Yuan C., Yin G. (2005). Numerical method for stationary distribution of stochastic differential equations with Markovian switching. J. Comput. Appl. Math..

[35] Kanehisa M., Goto S., Kawashima S., Okuno Y., Hattori M. (2004). The KEGG resource for deciphering the genome. Nucleic Acids Res..

[36] Kumar S., Stecher G., Li M., Knyaz C., Tamura K. (2018). MEGA X: Molecular evolutionary genetics analysis across computing platforms. Mol. Biol. Evol..

[37] Bastías D.A., Gianoli E., Gundel P.E. (2021). Fungal endophytes can eliminate the plant growth-defence trade-off. New Phytol..

[38] Xia C., Li N.N., Zhang Y.W., Li C.J., Zhang X.X., Nan Z.B. (2018). Role of *Epichloë* endophytes in defense responses of cool-season grasses to pathogens: A review. Plant Dis..

[39] Bastias D.A., Martınez-Ghersa M.A., Ballare C.L., Gundel P.E. (2017). *Epichloë* fungal endophytes and plant defenses: Not just alkaloids. Trends Plant. Sci..

[40] Swarthout D., Harper E., Judd S., Gonthier D., Shyne R., Stowe T., Bultman T. (2009). Measures of leaf-level water-use efficiency in drought stressed endophyte infected and non-infected tall fescue grasses. Environ. Exp. Bot..

[41] Ponce M.A., Bompadre M.J., Scervino J.M., Ocampo J.A., Chaneton E.J., Godeas A.M. (2009). Flavonoids, benzoic acids and cinnamic acids isolated from shoots and roots of Italian ryegrass (*Lolium multiflorum* Lam.) with and without endophyte association and arbuscular mycorrhizal fungus. Biochem. Syst. Ecol..

[42] Bastías D.A., Martínez-Ghersa M.A., Newman J., Card S.D., Mace W.J., Gundel P.E. (2018). The plant hormone salicylic acid interacts with the mechanism of anti-herbivory conferred by fungal endophyte in grasses. Plant Cell Environ..

[43] Hou W.P., Wang J.F., Christensen M.J., Liu J., Zhang Y.Q., Liu Y.L., Cheng C. (2021). Metabolomics insights into the mechanism by which *Epichloë gansuensis* endophyte increased *Achnatherum inebrians* tolerance to low nitrogen stress. Plant Soil.

[44] Khan A., Bassett S., Voisey C., Gaborit C., Johnson L., Christensen M., McCulloch A., Bryan G., Johnson R. (2010). Gene expression profiling of the endophytic fungus *Neotyphodium lolii* in association with its host plant perennial ryegrass. Australas. Plant Path..

[45] Chen N., He R.L., Chai Q., Li C.J., Nan Z.B. (2016). Transcriptomic analyses giving insights into molecular regulation mechanisms involved in cold tolerance by *Epichloë* endophyte in seed germination of *Achnatherum inebrians*. Plant Growth Regul..

[46] Dinkins R.D., Nagabhyru P., Graham M.A., Boykin D., Schardl C.L. (2017). Transcriptome response of *Lolium arundinaceum* to its fungal endophyte *Epichloë coenophiala*. New Phytol..

[47] Dinkins R.D., Nagabhyru P., Young C.A., West C.P., Schardl C.L. (2019). Transcriptome analysis and differential expression in tall fescue harboring different endophyte strains in response to water deficit. Plant Genome.

[48] Ambrose K.V., Belanger F.C. (2012). SOLiD-SAGE of endophyte-infected red fescue reveals numerous effects on host transcriptome and an abundance of highly expressed fungal secreted proteins. PLoS ONE.

[49] Amalric C., Sallanon H., Monnet F., Hitmi A., Coudret A. (1999). Gas exchange and chlorophyll fluorescence in symbiotic and non-symbiotic ryegrass under water stress. Photosynthetica.

