# Supplementary material for: Vertically Transmitted Epichloë Systemic Endophyte Enhances Drought Tolerance of Achnatherum inebrians Host Plants through Promoting Photosynthesis and Biomass Accumulation
Source: J Fungi (Basel). 2022 May 16;8(5):512. doi: 10.3390/jof8050512 (PMC9144827; doi:10.3390/jof8050512)
Supplement: Supplementary file 1 [file jof-08-00512-s001.zip › jof-1716943-supplementary.pdf]

Supplementary Materials

Supplementary for Material and Methods

Sampling, DNA extraction, quantification and qualification

Total RNA was extracted from the 100 mg fresh leaves of *Epichloë* symbiotic (EI) and non-symbiotic (EF) drunken horse grass (DHG) plants using Trizol reagent as per the manufacturer's instructions (Invitrogen, USA). The purity and concentration of RNA was checked using a NanoPhotometer® spectrophotometer (IMPLEN, CA, USA), and the integrity of RNA was assessed using the RNA Nano 6000 Assay Kit of the Agilent Bioanalyzer 2100 system (Agilent Technologies, CA, USA). RNA concentration was measured using the Qubit® RNA Assay Kit in a Qubit®2.0 Fluorometer (Life Technologies, CA, USA).

cDNA library construction and RNA sequencing

Transcriptome analysis in this present study was performed in the Biomarker Technologies (Beijing, China). A total amount of 3 μg RNA per sample was used as input material for the preparations of RNA sample. Sequencing libraries were generated using the NEBNext®Ultra™ RNA Library Prep Kit for Illumina® (NEB, USA). Afterwards, the libraries were sequenced by Illumina HiSeq 2000 platform.

In brief, mRNA was purified from total RNA using poly-T oligo-attached magnetic beads, and then random hexamer primer and M-M uLV Reverse Transcriptase (RNase H-) were used for thr first strand cDNA synthesis. Second strand cDNA was synthesized using DNA Polymerase I and RNase H. Remaining overhangs were converted into blunt ends via exonuclease/polymerase activities. After adenylation of 3' ends of DNA fragments, NEBNext Adaptors with hairpin loop structures were ligated prior to hybridization. cDNA fragments of 150~200 bp in length were purified using the AMPure XP system (Beckman Coulter, Beverly, USA). Then 3 μl USER Enzyme (NEB, USA) was used with size-selected, adaptor-ligated cDNA at 37°C for 15 min followed by 5 min at 95°C before PCR. PCR was performed with Phusion High-Fidelity DNA polymerase, Universal PCR primers and Index (X) Primer. PCR products were purified (AMPure XP system) and library quality was assessed on the Agilent Bioanalyzer 2100 system (Agilent, USA).

Sequence filtering, assembly and annotation

The clustering of the barcoded samples was performed on a cBot Cluster Generation System using the TruSeq PE Cluster Kit v3-cBot-HS (Illumina) according to the manufacturer's instructions. After cluster generation, the library preparations were sequenced on an Illumina Hiseq 2000 platform and paired-end reads were generated. Raw data (raw reads) of fastq format were firstly processed through in-house perl scripts to remove low quality reads. Reads removing adapters and poly-Ns were obtained as the clean reads (=138.42 Gb, Q30 > 89.1%) with high quality. The Do novo transcriptome assembly of these clean reads into 92 964 unigenes (N50 of 1 677 bp) were accomplished using TRINITY (v2.11.0) program (Grabherr *et al*., 2011) with set parameters (min_kmer_cov set to 2). Clean data were mapped back onto the assembled transcriptome; read count for each gene was obtained from the mapping results.

Meta-analysis

Papers in journals were collected from the Web of Science and the China National Knowledge Infrastructure (CNKI) databases during July 2020. These papers were searched with the keywords "*Epichloë*/endophyte/photosynthesis". A total of 26 papers in English and 223 papers in Chinese were located, and then the following criteria were used to select the appropriate papers.

1) The host plants of are the cool-season grass species. Here, a total of 21 papers in English and 78 papers in Chinese were selected for the next selection, respectively.

2) The references of these selected papers were also checked to search for further appropriate papers. Eight additional papers in English were obtained for obtaining data.

3) The selected papers had to include the means of net photosynthetic rate for both the endophyte-

infected and endophyte-free grasses. Here a total of 45 papers including 14 papers in English and 31 papers in Chinese were selected for the present meta-analysis.

The extract values of these selected papers were obtained from the graph using GetData Graph Digitizer 2.22 (http://getdata-graph-digitizer.com/). A total of 277 observations of photosynthetic efficiency, 29 observations of water use efficiency and 22 observations of photochemical efficiency. The mean, standard deviations (= standard errors $\times\sqrt{n}$, and the unreported standard deviations was estimated with the 10% of the mean) and number of repetitions (n) of endophyte-infected (experimental group) and endophyte-free (control group) were extracted from the 45 selected papers. The meta-analysis was performed through the MetaWin software (version 2.1) according to Hedges *et al*. (1999), and the effects of *Epichloë* endophytes on the net photosynthetic efficiency were calculated through log of the response ratio (*lnR*) using the formula (Hedges *et al*., 1999). The variance of the natural logarithm of the response ratio (*lnR* (v)) was approximated by the equation [2].

$$lnR = \ln(\frac{\overline{X_e}}{\overline{X_C}})=\ln(\overline{X_e})\text{-}\ln(\overline{X_c}) \qquad [1]$$

Where $\overline{X_e}$ and $\overline{X_c}$ represent the mean net photosynthetic efficiency in the experimental group and control group, respectively.

$$lnR(v) = \frac{S_e^2}{n_t\bar{X}_e^2} + \frac{S_c^2}{n_c\bar{X}_c^2} \qquad [2]$$

Where n_e and n_c indicate the repetitions of the experimental group and control group, respectively, and S_e^2 and S_c^2 indicate the standard deviations of experimental group and control group, respectively (Gurevitch *et al*., 2001). The *lnR* mean of the effects of *Epichloë* endophytes presence on the net photosynthetic efficiency was calculated with the combination of *lnR* and *lnR(v)*, and the *lnR(v)* was weighted by the inverse variance of the *lnR* for each observation. The 95% bootstrap confidence intervals for the mean *lnR* were calculated by 9999 iterations of bootstrapping according to a previous study (Gurevitch *et al*., 2001).

Table S1: Selected unigenes associated with processes of photosynthesis and photosynthesis-antenna proteins identified in the RNA-seq analysis in the present study.

No.	Primers (5’-3’)	
	Forward	Reverse
c61885.graph_c1	GCAGGTGCGCATATTGTGTT	ACATGGAATGCGCCAAAACC
c59956.graph_c0	AACCGGGGTCGTGGTAAATC	GCAATAGAGAGCGAACGGGA
c51264.graph_c3	CTGGGGACCAATCACAATCCA	ATCCTGCAGCCGGAAATGAG
c47798.graph_c0	GTGAAAAAGGCTGCACCGAA	AAACCCAAGCATGGCAACAC
c57444.graph_c0	ACAAGATATCAGGCACCCCC	AGCAAACGAGAGGAGATGGG
c60128.graph_c1	ACAGCACGTACTTCCACGAC	GGCAGACGTTGTGAGAATGC
c54875.graph_c1	CATAGTGACGCTTGAGCCGA	ACAGCACGTACTTCCACGAC
c58037.graph_c0	GCCACGAAATAAGAGCGGTG	CACTCGTCGTGAAGCTGGTA
c51322.graph_c1	GTAGTTGTTCGTCGACACGC	TTGATCAGGACCCCAACACC
c47792.graph_c0	CTCAGGGACTGCTTTGACGA	GCCAAAGATCGATGGCGATG
c61616.graph_c4	GTCGGAAAGGTCACCGTCAA	TTCTCTGCTCAACTCGTCGG
c52166.graph_c0	ATGCGCCTGAAGACACCTAC	AAGGAAGGAACCCTCCGACT
c47702.graph_c0	GAGGTCGGACTTGGGGTAAG	CGACGTCTACATCCTCGACC
c45025.graph_c0	ATGTCTGTTCAGCAGGCGAG	TGGAGCATTAGGGCCTACCA
c51525.graph_c1	GTTGCACGGACATGCAGAAG	CGTAACAAAGCATGGAGCGG
c46095.graph_c0	GATCTTTGGCCACCCGTTCT	CGAGAGGGCTGTGTACCTTC
c57544.graph_c0	CACGACCACGTTGAGGAAGT	AGTTCTTCGACAACCCGACC
c19569.graph_c0	CCTCTAGCTCACGGTTCTTGG	GTTCCCTGGTGACTATGGGTG
c33081.graph_c0	CTGCTTGAGGCAACCTGCT	CTTGCCCTCGGCTCTCTTTG
c36282.graph_c0	GCGGTTCTTGGCGAATGTTT	ATACCTTGGTCCGCTGTCTG
c47622.graph_c0	CGAGAACCTTTCCGACCACA	ATCGATCCCCGTAGTCCACA
c56765.graph_c1	CTCCGGTCTTAGAGGAGCAA	CGATGATGGCAGGTTCTTCAC
c56765.graph_c3	CCTTGCCTAAACCTAACACAGC	GGTGTGGAGATGGAGAGAACAA
c58363.graph_c2	CTGACCACATCACTGACCCC	CCTCGTTGACGCCTCACTT
c60825.graph_c3	TCACTGGCAAAGGACCCATC	GAAAGCCCATGCGTTGTTGT
c64087.graph_c0	GTGAAATCGTCGACCCACTCT	TGAAGGAGATCAAGAACGGGC
c65061.graph_c0	GCAAGTTCTTCACCGCCTTG	TGGAGAACACACACGACACC
c60825.graph_c0	GATCTCAGGGAACACGCACC	GCTGACCCAGAGACTTTCGC
c60825.graph_c2	ATGAACCATCGGTGCCTGTT	AGGGCTAAGGCAACTCCAAC
c54664.graph_c2	CTTCGACCACCTTGACGACC	GCATGGTTCGACACACAAGAG
c47083.graph_c1	TCCATGCTCGGGTTCTTCATC	TGATCGACGGCGAGCTTACA
c47083.graph_c2	TCCAGGAACTTCTCCGTTGAG	GAGCTCGCGAAGTGGTATGG
c46715.graph_c0	AAGATTTCCCCCTCCCCGAT	TGCGTGTCAAGTCCCTTCTT

Table S2: The comparative statistics between RNA sequencing clean data and transcriptome assembly of *Epichloë* symbiotic (EI) and non-symbiotic (EF) *Achnatherum inebrians* plants under normal (CK), moderate (MD) and severe (SD) drought treatments

Samples	Clean Reads	Mapped Reads	Mapped Ratio
SD EI-1	22 052 990	14 876 490	67.46%
SD EI-2	21 226 790	13 771 783	64.88%
SD EI-3	22 465 222	15 571 287	69.31%
SD EF-1	27 212 504	19 301 399	70.93%
SD EF-2	26 178 474	18 146 737	69.32%
SD EF-3	39 651 336	26 870 420	67.77%
MD EI-1	22 110 964	15 393 963	69.62%
MD EI-2	23 504 857	16 490 267	70.16%
MD EI-3	25 358 881	16 704 233	65.87%
MD EF-1	30 351 249	20 040 455	66.03%
MD EF-2	21 265 647	14 344 483	67.45%
MD EF-3	24 703 922	16 965 005	68.67%
CK EI-1	25 570 343	17 215 380	67.33%
CK EI-2	22 544 277	15 544 096	68.95%
CK EI-3	23 406 498	15 850 119	67.72%
CK EF-1	25 619 757	17 562 006	68.55%
CK EF-2	22 198 415	15 062 681	67.85%
CK EF-3	37 489 169	25 091 217	66.93%
Total	462 911 295	314 802 021	68.00%

Table S3: Length distribution of transcripts and unigenes of *Achnatherum inebrians* plants

Length distribution of transcripts and Unigenes	Transcript	Unigene
200-300	36 501 (14.87%)	29 529 (31.76%)
300-500	37 129 (15.13%)	22 216 (23.90%)
500-1000	52 080 (21.22%)	19 511 (20.99%)
1000-2000	60 242 (24.54%)	10 940 (11.77%)
≥2000	59 519 (24.25%)	10 768 (11.58%)
Total Number	245 471	92 964
Total Length	338 709 144	80 431 027
N50 Length	2 230	1 677
Mean Length	1379.83	865.18

Table S4: Unigenes statistics of *Achnatherum inebrians* plants transcriptome against eight different public databases.

Annotated Database	Annotated Number	Percentage (%)
COG Annotation	12 338	13.27
GO Annotation	25 308	27.22
KEGG Annotation	12 455	13.40
KOG Annotation	22 499	24.20
Pfam Annotation	27 008	29.05
Swissprot Annotation	20 642	22.20
eggnog Annotation	38 995	41.95
Nr Annotation	36 831	39.62
All Annotated	42 618	45.84

Table S5: The ANOVA table showing the effects of symbiosis status and soil moisture levels on the growth partners, biomass and photosynthetic indices of *Achnatherum inebrians* plants

Response variable	Treatments	*df(n,d)*	F	*P*-value
Plant height (cm)	Symbiosis	1,48	961.156	**<0.001**
(*n* = 9)	Soil moisture	2,48	101.071	**<0.001**
	Symbiosis x Soil moisture	2,48	17.274	**<0.001**
Shoot fresh weight (g)	Symbiosis	1,48	273.880	**<0.001**
(*n* = 9)	Soil moisture	2,48	504.240	**<0.001**
	Symbiosis x Soil moisture	2,48	23.610	**<0.001**
Root fresh weight (g)	Symbiosis	1,48	48.470	**<0.001**
(*n* = 9)	Soil moisture	2,48	555.300	**<0.001**
	Symbiosis x Soil moisture	2,48	8.830	**<0.001**
Shoot dry weight (g)	Symbiosis	1,48	178.356	**<0.001**
(*n* = 9)	Soil moisture	2,48	14.707	**<0.001**
	Symbiosis x Soil moisture	2,48	3.470	0.076
Root dry weight (g)	Symbiosis	1,48	21.966	**<0.001**
(*n* = 9)	Soil moisture	2,48	59.940	**<0.001**
	Symbiosis x Soil moisture	2,48	1.446	0.246
Intercellular CO_2	Symbiosis	1,48	20.156	**<0.001**
concentration	Soil moisture	2,48	37.710	**<0.001**
(CO_2 $mmol^{-1}$) (*n* = 9)	Symbiosis x Soil moisture	2,48	9.777	**<0.001**
Transpiration rate	Symbiosis	1,48	4.393	**0.041**
(mmol H_2O m^{-2} s^{-1})	Soil moisture	2,48	49.761	**<0.001**
(*n* = 9)	Symbiosis x Soil moisture	2,48	16.401	**0.003**
Stomatol condutance	Symbiosis	1,48	7.303	**0.009**
(mmol H_2O m^{-2} s^{-1})	Soil moisture	2,48	78.276	**<0.001**
(*n* = 9)	Symbiosis x Soil moisture	2,48	6.117	**0.004**

Note: Statistically significant effects are highlighted in bold.

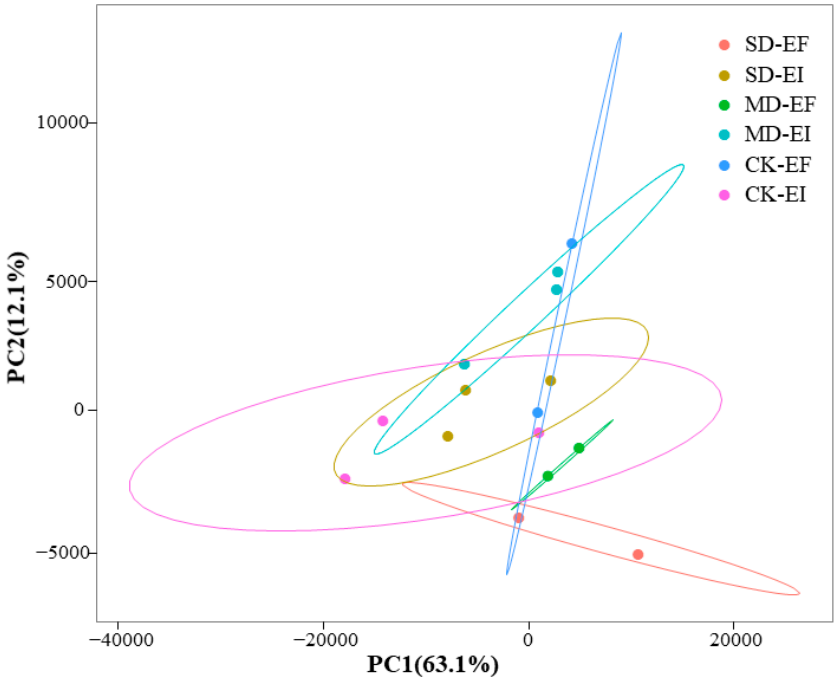

Figure S1: Principal component analysis (PCA) of all unigenes from *Epichloë* symbiotic (EI) and non-symbiotic (EF) *Achnatherum inebrians* plants under normal (CK), moderate (MD) and severe (SD) drought treatments.

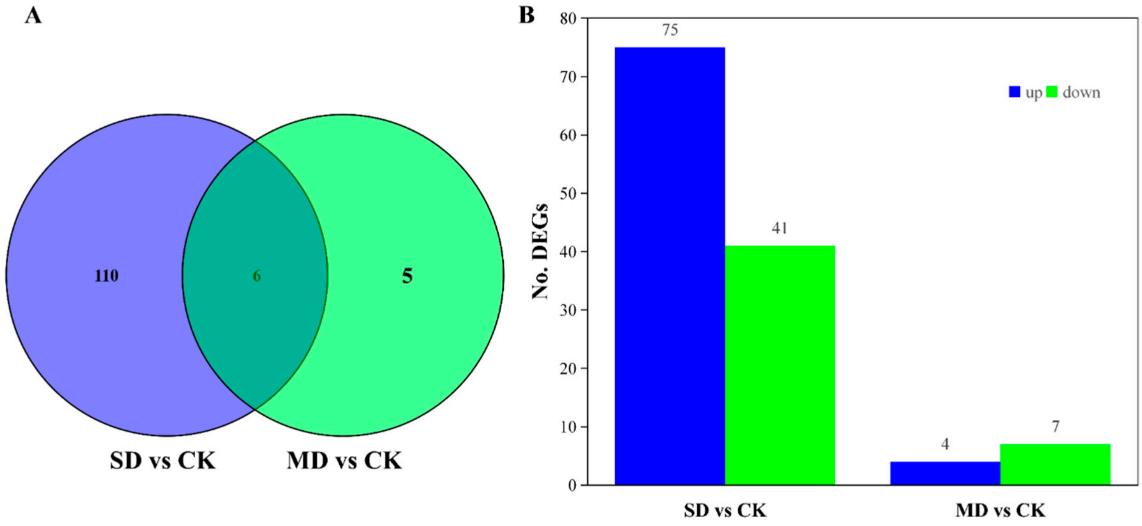

Figure S2: The number of up- and down-regulated differently expressed unigenes (DEGs, FC>1.5) of *Achnatherum inebrians* plants under the several (SD) and moderate drought (MD) treatments compared to the normal (CK) treatment (DEGs in endophyte-infected plants versus endophyte-free plants).

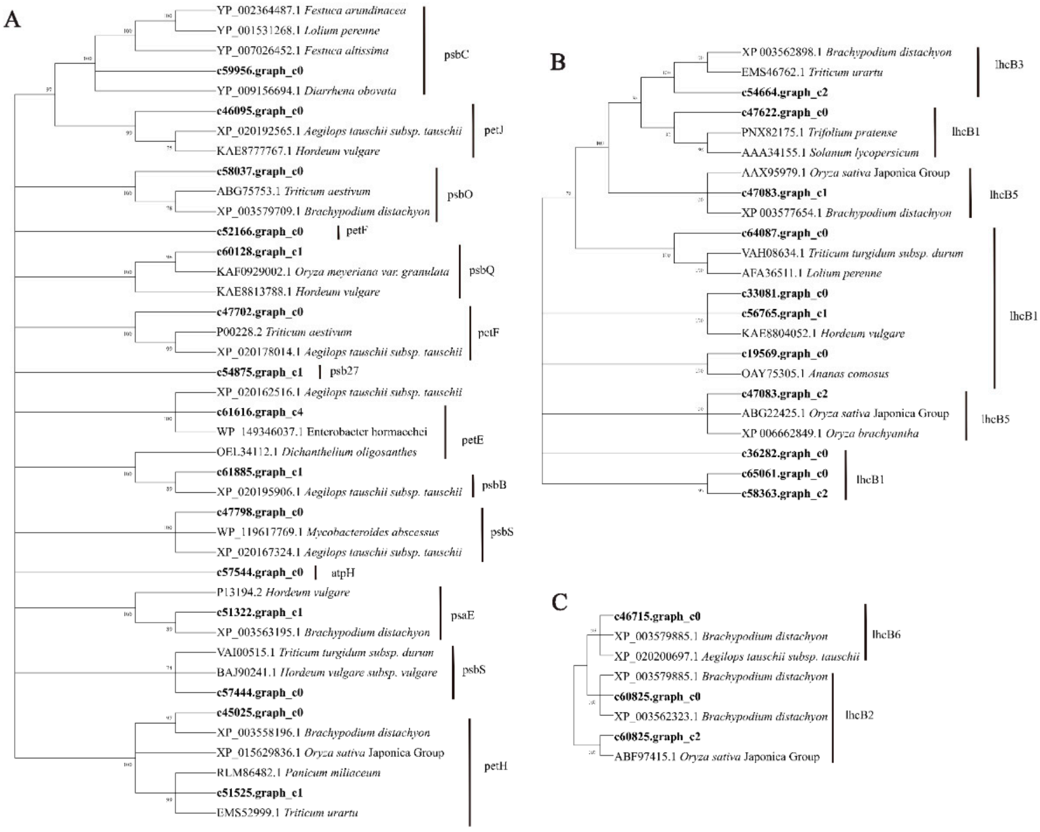

Figure S3. The Neighbor-Joining (NJ) tree showing the amino acid sequences of differently expressed genes (DEGs) associated with photosynthesis (**A**) and photosynthesis-antenna proteins (**B**,**C**) of *Achnatherum inebrians* plants. All bootstrap values >70% are shown (1000 replicates). Numbers above branches indicate the bootstrap values of the maximum likelihood analysis.

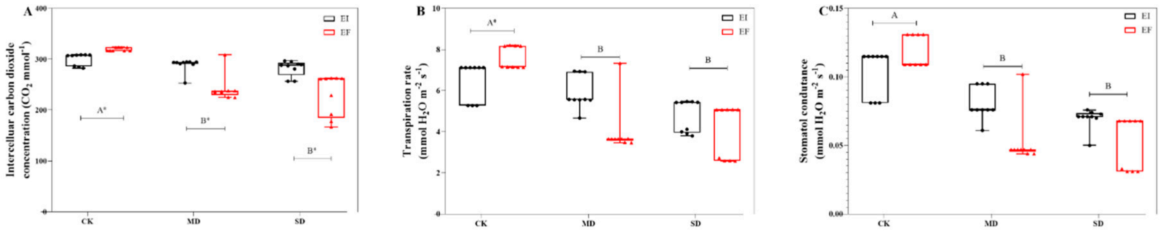

Figure S4: The intercellular carbon dioxide concentration (A), transpiration rate (B) and stomatal conductance (C) of *Epichloë* symbiotic (EI) and non-symbiotic (EF) *Achnatherum inebrians* plants under normal (CK), moderate (MD) and severe (SD) drought treatments. The asterisk (*) means significant difference at $P<0.05$ (independent t-test) between and EI and EF plants at corresponding water content at 0.05 level. The A and B mean significant differences among corresponding water content at 0.05 level.

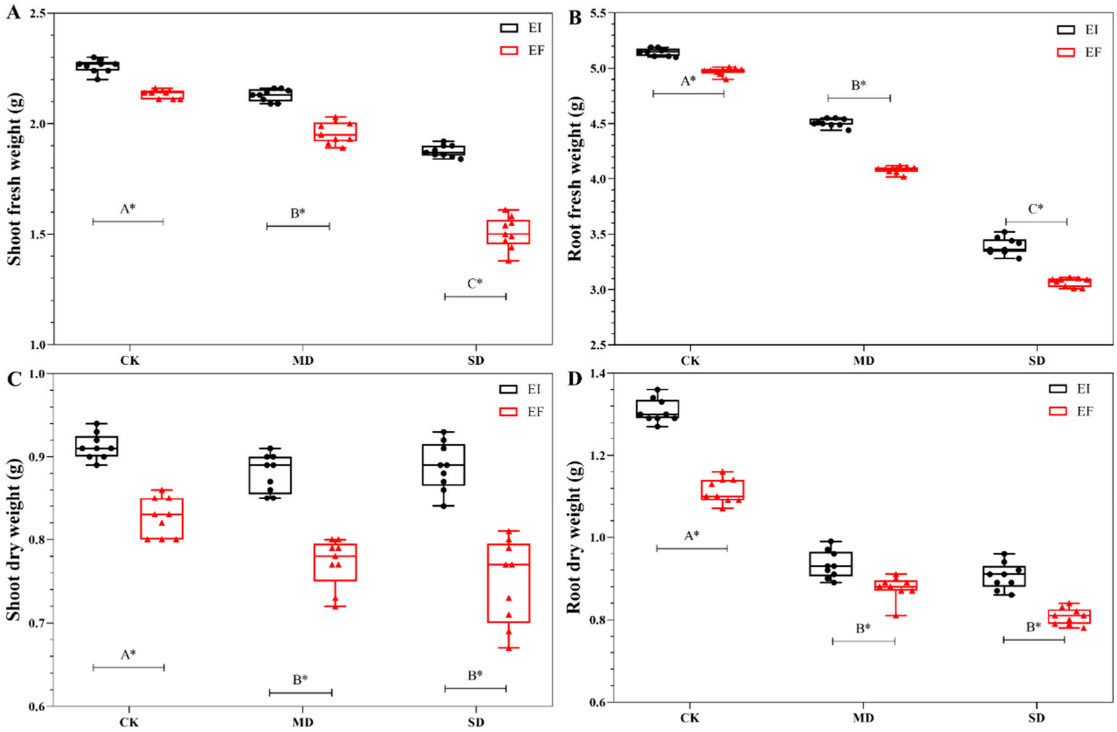

Figure S5: The fresh weight of shoot (a) and root (b), and dry weight of shoot (c) and root (d) of *Epichloë* symbiotic (EI) and non-symbiotic (EF) *Achnatherum inebrians* plants under normal (CK), moderate (MD) and severe (SD) drought treatments. The asterisk (*) means significant difference at $P<0.05$ (independent t-test) between and EI and EF plants at corresponding water content at 0.05 level. The A, B and C mean significant differences among corresponding water content at 0.05 levels.
